# Effect of narrowband ultraviolet B (311 nm) exposure on skin carcinogenesis in Wistar rats

**DOI:** 10.5455/javar.2024.k861

**Published:** 2024-12-29

**Authors:** Roro Inge Ade Krisanti, Septelia Inawati Wanandi, Puspita Eka Wuyung, Aida S. D. Hoemardani

**Affiliations:** 1 Doctoral Program in Biomedical Sciences, Faculty of Medicine, Universitas Indonesia, Jakarta, Indonesia; 2 Department of Dermatology and Venereology, Faculty of Medicine, Universitas Indonesia, Jakarta, Indonesia; 3 Department of Biochemistry and Molecular Biology, Faculty of Medicine, Universitas Indonesia, Jakarta, Indonesia; 4 Center of Hypoxia and Oxidative Stress Studies, Faculty of Medicine, Universitas Indonesia, Jakarta, Indonesia; 5 Molecular Biology and Proteomic Core Facilities, Indonesian Medical Education and Research Institute, Faculty of Medicine, Universitas Indonesia, Jakarta, Indonesia; 6 Department of Pathological Anatomy, Faculty of Medicine, Universitas Indonesia, Jakarta, Indonesia

**Keywords:** Narrowband UVB, Skin carcinogenesis, Wistar rat

## Abstract

**Objective:**

The aim of this study is to determine narrowband UVB (NB-UVB) irradiation’s effect on the promotion of skin cancer, particularly its effect on DNA damage, oxidative stress, inflammation, and histological changes in Wistar rat skin.

**Materials and Methods:**

Wistar rats were selected for this study and randomly divided into control, dimethylbenzanthracene (DMBA), and DMBA+NB-UVB groups. The rats were given a single dose of DMBA and exposed to NB-UVB 3 times a week for 10 weeks. The radiation dose started with 1 minimal erythema dose, which is equivalent to 3.192 J/cm². In the 11th week, analysis on cyclobutene pyrimidine dimer (CPD), malondialdehyde (MDA), nuclear factor kappa-B (NFκB), inflammatory cytokines, and histopathology examination of the skin tissue was conducted.

**Results:**

Higher CPD, MDA, NFκB, tumor necrosis factor a (TNF-a), interleukin (IL)-6, IL-11, IL-10, and IL-12 levels in rats exposed to DMBA+NB-UVB for 10 weeks compared to control and DMBA groups. Macroscopic examination presented erythema, skin thickening, desquamation, ulcer, and crust. Histopathology examination showed hyperkeratosis, acanthosis, atypical keratinocytes, irregular arrangement of the basement membrane, and inflammatory cell infiltration in the DMBA+NB-UVB group.

**Conclusion:**

This research has shown that 10 weeks of a combination of DMBA and NB-UVB irradiation induced DNA damage, oxidative stress, inflammation, and histological changes in the Wistar rat skin.

## Introduction

Cutaneous squamous cell carcinoma (cSCC) is a common nonmelanoma skin cancer (NMSC), comprising approximately 20% of all cutaneous neoplasms [1,2]. cSCC demonstrates a complex clinical course, evolving from precursor lesions such as actinic keratosis to squamous cell carcinoma *in situ*, invasive cSCC, and, ultimately, metastatic cSCC [3]. Ultraviolet B radiation (UVB) is the primary risk factor for SCC. Cumulative exposure to ultraviolet light over a lifetime significantly contributes to cSCC risk [4–6]. The incidence of cSCC exhibits significant geographic variability and has persistently increased by over 50% in the past three decades [2]. Surgery remains the mainstay for treating cSCC; however, scar formation and functional limitations after surgery are still a challenge considering the anatomical site of the lesion, which is often found in the exposed area. Nonsurgical options have limitations [7], and with the increasing incidence of nonmelanoma skin cancer in Indonesia, preventive efforts against cSCC are crucial.

Comprehensive research to elucidate the underlying mechanisms of carcinogenesis events is essential to devise effective cSCC prevention and therapeutic strategies. A suitable animal model is beneficial for gaining deeper insights into disease biology, thereby increasing research success [1]. An animal model seeks to emulate human conditions or diseases. Though they may not entirely mimic all variables seen in human disease, they offer valuable insights into molecular and cellular aspects [3]. Research on cSCC carcinogenesis using animal models of transgenic mice with certain strands has been widely conducted. However, in Indonesia, the scarcity of animal models with certain strains remains a major challenge and limits experimental options. The Wistar rat is a promising experimental model because it has several advantages, including its wide availability and good genetic background characteristics.

The classic mouse model for studying SCC involves two-stage chemical carcinogenesis. Using a single topical dose of dimethylbenzanthracene (DMBA), followed by repeated administration of 12-O-tetradecanoylphorbol-13-acetate (TPA), can promote the growth of papillomas that can develop into SCC, which can then metastasize to distant sites [8,9]. Although this method has been widely applied, on the other hand, the induction of carcinogenesis with UV in animal models is biologically considered to be more similar to carcinogenesis in humans, considering that 90% of NMSC in humans are estimated to be caused by excessive exposure to UV radiation [10].

UVB (280–320 nm) exposure plays a role in photocarcinogenesis as an initiator and promoter [11]. It directly damages DNA and generates reactive oxygen species (ROS), leading to oxidative stress and inflammation. Additionally, UVB exposure activates signaling pathways that promote inflammation, immunosuppression, and cell proliferation, further encouraging carcinogenesis [12]. Notably, narrowband UVB (NB-UVB) is widely used for treating various skin conditions such as psoriasis, atopic dermatitis, and vitiligo due to its efficacy and safety profile compared to broadband UVB (BB-UVB) [13–15]. However, despite these therapeutic benefits, NB-UVB has been implicated as a potential carcinogen. A cohort study in Finland by Åkerla et al. [16] found an increased incidence of skin cancer in psoriasis and atopic dermatitis patients undergoing phototherapy with NB-UVB radiation (TL-01). Other studies in genetically modified mice have shown that prolonged exposure to NB-UVB can induce DNA damage and mutations similar to those caused by natural UVB, thereby increasing the risk of skin cancer [17]. Additionally, research by Yogianti et al. [18] demonstrated that NB-UVB exposure caused tumor development on mice’s skin by inducing *p53* mutations through cyclobutene pyrimidine dimer (CPD) formation. This emphasizes the importance of understanding NB-UVB’s effects on skin carcinogenesis.

Some previous studies have highlighted the significance of the promotion phase in cSCC development. The promotion phase of SCC involves intricate molecular mechanisms and inflammatory cascades. These factors contribute to DNA damage, oxidative stress, and inflammatory responses, thereby fostering tumor progression [12]. In this study, we hypothesize that chronic exposure to NB-UVB after DMBA initiation will exacerbate DNA damage and oxidative stress and increase inflammatory responses, which play a role in skin carcinogenesis promotion of Wistar rat skin. This hypothesis is based on the understanding that UVB can cause direct DNA damage and modulate inflammatory pathways, leading to tumor initiation and promotion [12].

The novelty of our research lies in using a single dose of DMBA combined with chronic NB-UVB exposure to promote skin carcinogenesis in Wistar rats, a readily accessible and common model used in Indonesia. Unlike traditional models, which often rely on genetically modified animals or multistage chemical carcinogenesis protocols, this research focuses on the role of NB-UVB in promoting skin cancer after DMBA initiation. The study provides a comprehensive evaluation of how NB-UVB influences skin carcinogenesis through mechanisms of DNA damage and inflammatory responses that then trigger histological alterations, offering a perspective on the interaction between NB-UVB exposure and chemical initiation in skin cancer development. In this study, we examined parameters such as CPD, malondialdehyde (MDA), NFkB, and inflammatory cytokines (TNF-α, IL-6, IL-11, IL-10, and IL-12) to understand the molecular and histological changes induced by these exposures, making it a practical and relevant choice for skin cancer research in Indonesia.

## Materials and Methods

### Ethical approval

The protocol for this animal study was accepted by the Ethical Committee, Faculty of Medicine, Universitas Indonesia, Jakarta (Protocol Number: 23-04-0480, Date of Approval: 01 May 2023).

### Study design

The study was an experiment involving Wistar rats. The sample size was determined using the resource equation approach for a one-way ANOVA design, ensuring the degrees of freedom (DFs) stayed within an acceptable range (10–20) [19,20]. By substituting the minimum (10) and maximum (20) DF values in the calculation, each group should comprise 4–6 animals. Consequently, the total sample size required to maintain a DF value between 12 and 20 was 12–18 animals. Based on this calculation, the study employed 18 rats


$$n\;(\min\;\mathrm{or}\;\max)\;=\;\frac{\mathrm{DF}\;(\min\;\mathrm{or}\;\max)}{k}\;+\;1$$

n = number of animals per group

DF = Degree of freedom

k = number of groups.

### Animals and treatment

A total of 18 Wistar rats were selected for this study. The rats were randomly divided into 3 groups, consisting of a control, DMBA, and DMBA + NB-UVB group. The rats were obtained from PT. Bio Farma, Bandung Barat, Jawa Barat, Indonesia. The age of the rats in this study ranged from 10 to 12 weeks. Rats were placed at the Animal Research Facilities IMERI FKUI, Jakarta, Indonesia. The rats were housed in cages with access to food and water. The light was maintained for a 12-hour light and dark cycle with no access to direct sunlight.

The rats were acclimated for 7 days prior to the experiment. The dorsal area was shaved every five days in an area measuring 3.5 × 5 cm. Before shaving, rats were injected intra-peritoneally with ketamine (40–90 mg/kg BW) and xylazine (5–10 mg/kg BW) as an anesthetic. Next, a single dose of 1% (w/v) DMBA (0.01 gm of DMBA diluted in 1 ml of absolute acetone) was applied to the shaved area of the rat’s back using a micropipette.

Minimal erythema dose (MED) was determined prior to DMBA and NB-UVB induction. The minimal erythema dose obtained was 3.192 J/cm² (20 min). One week after DMBA application, 311-nm NB-UVB light was exposed to the DMBA+NB-UVB group 3 times a week for 10 weeks. The radiation dose begins with a duration of 20 min (1 MED), which is equivalent to 3.192 J/cm² and is increased to 30 min (1.5 MED) at the beginning of the 4th week. The radiation dose of 1.5 MED was maintained from the fourth to sixth week. Entering the seventh week, the radiation dose was again increased to 40 min (2 MED) and maintained until the end of the 10th week. Excision of skin tissue is carried out on the last day after radiation in the 10th week.

UV devices containing UV-B Narrowband TL 100W/01 SLV/10 (Phillips Ltd., London, United Kingdom) were used as the UV source. In total, four UVB Narrowband TL emitted irradiance of 0.00266 mW/cm^2^ (intensity) measured using the UV light meter Lutron YK-35UV (Lutron Electronic Enterprise Co., Ltd, Taipei City, Taiwan).

### Measurement of CPD level

DNA extracted from rat skin tissue served as the sample for quantifying CPD levels. Total DNA isolation employed a commercially available DNA isolation kit [Zymo Research, D4069], adhering to the manufacturer’s protocol. CPD level determinations were executed on 50 μl of 4-μg/ml DNA samples using the indirect ELISA [Abcam, ab242297] by the manufacturer’s recommended procedures.

### Skin tissue homogenate

Skin samples were homogenized with 0.01-M phosphate buffer saline, pH = 7.4 (1:9 w/v). Subsequently, the homogenate was centrifuged at 5,000 × gm for 5 minutes at 4°C. Then, the supernatant was used for research parameters analysis, including MDA, nuclear factor kappa-B (NFκB)-p-65, and inflammatory factors (TNF-a, IL-6, IL-10, IL-11, and IL-12).

### Measurement of MDA level

The quantification of MDA levels in rat skin homogenates was performed using Will’s method, predicated upon the interaction between MDA and thiobarbituric acid (TBA). Subsequently, MDA within the sample underwent a reaction with TBA, yielding a thiobarbituric acid reactive substance (TBARS), whose concentration was assessed spectrophotometrically at 530 nm.

### Measurement of NFκB (p-65) and inflammatory factors

The levels of NFκB (p-65) [Finetest, ER0033] and inflammatory factors, namely, TNF-a [Elabscience, E-EL-R2856], IL-6 [Finetest, ER0042], IL-10 [Finetest, ER0033], IL-11 [Finetest, ER0093], and IL-12 [Finetest, ER1086], were measured using the sandwich ELISA method as per the manufacturer’s instructions. To ensure the accuracy of the results, the concentrations of these NFκB and inflammatory factors were adjusted to the total protein content of each sample, which was determined using the Bradford method [BioRad, 5000006].

### Histopathology analysis

The excised skin tissue is fixed in 10% formalin and then dipped in alcohol and xylol solution. The tissue is then placed in a paraffin solution and arranged into a cassette. The paraffin hardens until a block forms. The paraffin block was then sliced using a microtome. Tissue that has been sliced is taken using a glass object. The tissue was stained using hematoxylin and eosin staining. Skin tissue preparations that had been stained with HE were then observed under a Leica DM750 microscope. Each skin tissue preparation was then photographed using a Leica MC190 HD Microscope Camera in 5 fields of view with 10× and 40× magnification. The images of the skin tissue preparations obtained were then analyzed descriptively.

### Statistical analysis

Statistical analysis employed ANOVA and Tukey’s test for normally distributed data. For nonnormally distributed data, we used the Kruskal–Wallis test followed by Dunn’s test. Data processing was conducted using GraphPad Prism software version 10.0.

## Results

### NB-UVB exposure induced DNA damage and oxidative stress

**Figure 1.**
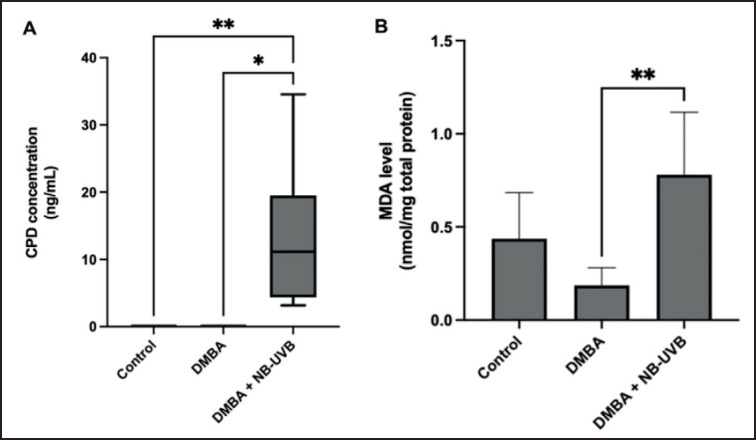
The effect of NB-UVB exposure on CPD and MDA levels in rat skin. NB-UVB exposure increased CPD (A) and MDA (B) levels. CPD data were analyzed using the Kruskal–Wallis followed by Dunn’s test. MDA data were analyzed using ANOVA followed by Tukey’s as post hoc test (*p < 0.05, **p < 0.01, and ***p < 0.001).

NB-UVB exposure particularly increases DNA damage by forming CPD. As depicted in Figure 1A, 10 weeks of NB-UVB exposure following DMBA administration significantly increased CPD levels in rats’ skin compared to the control and DMBA-only groups. In addition, this study evaluated the skin’s oxidative stress status following NB-UVB exposure through MDA-level assessment. Results revealed significantly higher MDA levels in rats exposed to DMBA and NB-UVB for 10 weeks compared to DMBA-only groups (Fig. 1B).

### NB-UVB exposure induced the expression of NFκB and inflammatory factors

**Figure 2.**
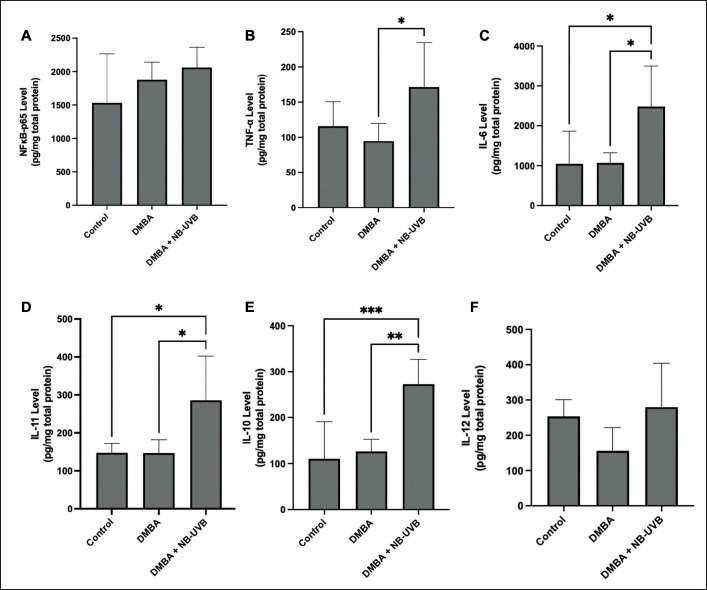
The effect of NB-UVB exposure on NFκB (A), TNF-α (B), IL-6 (C), IL-11 (D), IL-10 (E), and IL-12 (F) levels in rat skin. The data are presented as Mean ± SEM (ANOVA, Tukey’s, *p < 0.05, **p < 0.01, and ***p < 0.001).

NB-UVB exposure is recognized to influence NFκB expression. Although not statistically significant, the results revealed an increase in NFκB levels in the rat skin following ten weeks of NB-UVB exposure after DMBA administration compared to the control and DMBA-only groups (Fig. 2A). In addition, various inflammatory factors, including TNF-α, IL-6, and IL-11, are implicated in promoting skin photocarcinogenesis. The findings indicated that skin samples from the group exposed to NB-UVB for 10 weeks post-DMBA administration exhibited elevated TNF-α levels compared to the control and DMBA-only groups (Figure 2B). Similarly, this group also demonstrated significantly heightened levels of IL-6 (Fig. 2C) and IL-11 (Fig. 2D) compared to the control and DMBA-only groups. Additionally, following 10 weeks of NB-UVB exposure post-DMBA administration, a significant increase in IL-10 levels (Fig. 2E) with marginal elevation in IL-12 levels was observed, compared to control and DMBA-only groups (Fig. 2F), indicating a potential shift in the immune response.

### NB-UVB exposure causes skin erythema and histological damage

**Figure 3.**
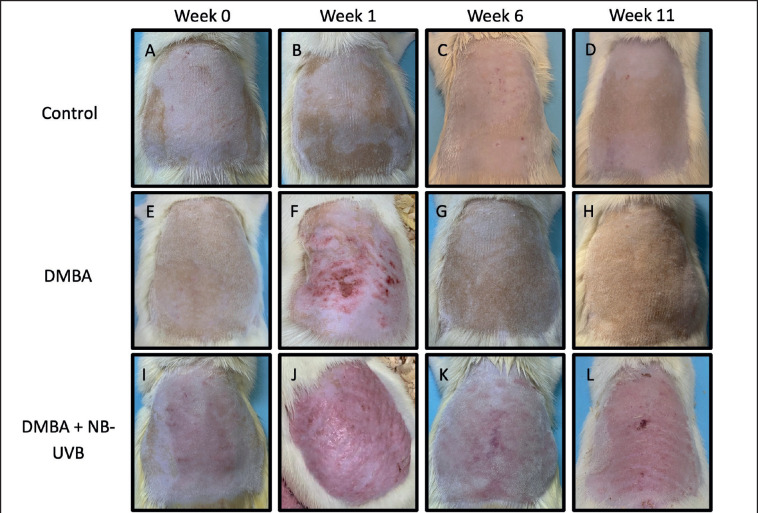
Macroscopic evaluation of the dorsal skin of Wistar rats. (A–D) Representative macroscopic of non-DMBA and non-NB-UVB irradiated control group. (E–H) Representative macroscopic of DMBA group. (I–L) Representative macroscopic of DMBA and NB-UVB irradiated group. DMBA was applied in week 0 for DMBA and DMBA + NB-UVB group (photo was taken before topical DMBA was applied). DMBA + NB-UVB group started irradiated with NB-UVB on the first day of week 1.

**Figure 4.**
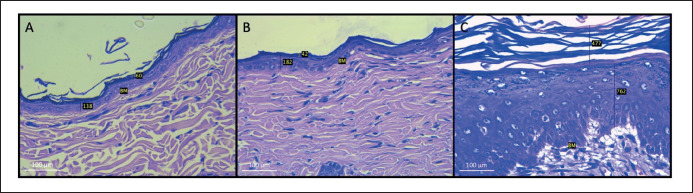
Histopathology examination of the dorsal skin of Wistar rats (H&E staining). (A) Histopathology of non-DMBA and non-NB-UVB irradiated control group. (B) Histopathology of DMBA group. (C) Histopathology of DMBA and NB-UVB irradiated group. Histopathology examination was observed in week 11 with 40× magnification.

In Figure 3, it can be seen that macroscopically, experimental animals that were given DMBA and NB-UVB exposure for 10 weeks showed erythematous skin accompanied by thickening of the skin, desquamation, ulcer, and crust. Meanwhile, in the control group and the group given DMBA-only, there was no visible redness or thickening of the skin. The histopathological analysis of skin tissue of the DMBA and NB-UVB groups showed hyperkeratosis, epidermal thickening, atypical keratinocytes, irregular arrangement of the basement membrane layer, and inflammatory cell infiltration. In the control and DMBA groups, there was no hyperkeratosis or epidermal thickening, along with an orderly arrangement of the basement membrane (Fig. 4).

## Discussion

Numerous studies have investigated animal models of skin carcinogenesis in cSCC, including the complete and two-stage methods. In the complete skin carcinogenesis model, tumor development occurs without additional carcinogens after high-dose exposure or repeated lower doses [3,21]. Differentiating between initiation and promotion stages complicates interpretation. On the other hand, the two-stage model distinctly separates these stages [21]. Typically, the model begins with administering chemically carcinogenic DMBA, followed by tumor promotion agents such as TPA. Despite extensive application, the DMBA/TPA model is less representative of human events, primarily induced by UV light exposure [1,3]. Our study aims to provide an overview of how exposure to NB-UVB light induces the promotion phase of a two-stage skin carcinogenesis model in Wistar rats after topical administration of DMBA.

Topical DMBA was used in this study. Factors affecting topical drug administration consist of skin microflora, skin pH, skin surface lipids, temperature, blood flow, skin disease, skin adnexa (hair follicles and sweat glands), skin metabolism, age, race, and individual variations [22]. The aim of applying a single dose of DMBA in this study was as an initiator, followed by administering NB-UVB as done by Kapadia et al. [23], which we have modified.

NB-UVB, situated within the 311-nm wavelength of the BB-UVB spectrum [24], has garnered considerable attention as a potential carcinogen, particularly in the progression of skin cancers such as cSCC. Our hypothesis proposed that NB-UVB exposure, in combination with DMBA, would exacerbate DNA damage and oxidative stress to initiate and promote skin carcinogenesis. Our findings support this hypothesis, as evidenced by the significant increase in CPD formation (Fig. 1A) and MDA levels (Fig. 1B) in the rat skin subjected to DMBA and NB-UVB exposure over 10 weeks, compared to the control group and those treated solely with DMBA. NB-UVB possesses adequate energy to facilitate the formation of covalent bonds between adjacent pyrimidine bases (such as thymine or cytosine) within the DNA, resulting in CPD formation [25,26]. The study by Carpenter et al. [27] and Toriyama et al. [28] found that NB-UVB exposure can elevate CPD formation in the human keratinocyte and BALB/cAnN skin mice, respectively [27,28]. Previous studies have also reported the carcinogenic potential of NB-UVB via CPD production. For instance, Kunisada et al. [17] demonstrated that exposure to NB-UVB resulted in greater CPD formation and increased tumor formation in C57BL/6J mice, albino hairless, and genetically modified mice skin compared to BB-UVB exposure [17].

In addition to CPD, our study found significantly higher MDA levels in rat skin exposed to DMBA and NB-UVB for ten weeks compared to controls and DMBA alone (Fig. 1B). These may be due to UVB exposure increasing ROS [29,30] and depleting antioxidants in the skin [31–33]. When skin molecules absorb UVB photons, they trigger photochemical reactions that actively produce ROS [29,30]. Additionally, frequent UVB exposure can deplete the skin’s antioxidant system, exacerbating oxidative stress and leading to MDA formation as a marker of this process [34,35]. Our findings were in line with Carrara et al. [35], which showed that chronic UVB exposure increased MDA and disrupted the redox balance in the skin of hairless mice (HRS/J), as indicated by decreased levels of GSH and catalase. The study also established a correlation between this redox imbalance and the development of skin cancer.

The accumulation of DNA damage and oxidative stress leads to mutations in critical genes responsible for cell growth and proliferation, including tumor suppressor genes and oncogenes [26,36,37]. UVB radiation can stimulate the production of growth factors, cytokines, and other signaling molecules that support the survival and proliferation of mutated cells. This promotion phase involves complex interactions between UV-induced signaling pathways, inflammatory responses, and alterations in the microenvironment of the skin [38]. Moreover, UVB radiation-induced inflammation can elucidate the promotion of carcinogenesis by generating ROS and activating pro-inflammatory pathways that further damage DNA and promote cell proliferation [12,38].

Our study found that NB-UVB exposure increases NFκB levels (Fig. 2A) and pro-inflammatory cytokines (TNF-α, IL-6, and IL-11) (Figs. 2B–D), which are crucial in promoting skin carcinogenesis [12,39]. UVB rays primarily stimulate the skin to boost NFκB via ROS production. ROS alters inhibitor kappa-B (IκB), enabling NFκB to become active in the nucleus. Activation of NFκB extensively increases cytokine release, leading to heightened inflammation and the release of cell growth factors crucial for promoting cancer formation [12,39]. Additionally, chronic UVB exposure causes DNA damage in skin cells, further activating pathways that increase NFκB activation. Moreover, chronic UVB exposure indirectly triggers NFκB by releasing cytokines and growth factors from damaged skin cells, increasing inflammation and promoting nearby skin cancer development [12,39].

Exposure to UVB radiation also activates immune cells in the skin, such as dendritic cells and macrophages, thereby releasing various pro-inflammatory cytokines, including TNF-α [40], IL-6 [41], and IL-11 [12]. Pro-inflammatory cytokine secretion after UVB exposure can also originate from activating keratinocytes, the dominant epidermal cells [42]. TNF-α, IL-6, and IL-11 play a critical role in promoting the development and progression of cSCC by creating an inflammatory environment that supports the growth and survival of cancer [12,39]. TNF-α, particularly, has demonstrated the ability to stimulate keratinocyte proliferation, potentially contributing to cSCC development, while IL-6 and IL-11 can enhance cancer cell survival and migration, thereby bolstering cSCC growth [12,42].

Our study also found that IL-10 and IL-12 levels were affected by NB-UVB exposure in combination with DMBA (Figs. 2E and F). Elevated IL-10 levels potentially suppressed the immune response [12]. UVB exposure boosts IL-10 production by activating skin immune cells such as keratinocytes and dendritic cells. IL-10 serves as an anti-inflammatory cytokine, regulating immune responses and maintaining homeostasis. However, excessive IL-10 production in response to chronic UVB exposure may suppress the immune system’s ability to recognize and eliminate cancerous cells and promote cSCC development [12,39].

UVB exposure also impacts IL-12 expression in the skin. IL-12, chiefly generated by dendritic cells and macrophages, is essential in promoting Th1 immune responses and anti-tumor defenses [12,43]. UVB-induced DNA damage and oxidative stress can stimulate the production of IL-12, leading to the activation of cytotoxic T cells and natural killer cells, which are crucial for anti-tumor immune responses. However, the regulatory role of IL-12 in cSCC development is complex, as excessive IL-12 production may also contribute to chronic inflammation and tissue damage, potentially promoting tumor progression [12,39]. Overall, those findings support our hypothesis that NB-UVB exposure can modulate the immune response in the skin, which might trigger the promotion of skin carcinogenesis.

Macroscopically, rats in the NB-UVB and DMBA groups showed erythema, thickening of the skin, desquamation, ulcers, and crust. Similar results were demonstrated by Korinfsky et al. [44], who used Wistar rats and investigated animal skin that had been exposed to UVB and DMBA three times a week for 10 weeks. At the end of 10 weeks, dermatological lesions presented were erythema, desquamation, keratosis, and ulcer [44]. Concurrently, 24-week UVB radiation showed significantly higher tumor number and epidermal thickness [45].

During the promotion stage of carcinogenesis, as the DNA synthesis and inflammation increase, histopathological examination showed hyperplastic epidermis [21]. This is in accordance with our findings. In our study, the DMBA + NB-UVB group showed hyperkeratosis, parakeratosis, acanthosis, atypical keratinocytes, inflammatory cell infiltration, and irregular basement membrane. NB-UVB irradiation induced chronic inflammation, which may represent the promotion stage of carcinogenesis; however, not all chronic inflammation indicates this stage of carcinogenesis. Tumor promotion is characterized by higher proliferation of epidermal cells and inflammation [46].

The histopathological picture, squamous cell carcinoma apart from atypical keratinocytes, was found to be circles of “horn pearl” parakeratosis, atypical mitotic keratinocytes with dense and large nuclei that were pleomorphic and hyperchromatic, dyskeratotic keratinocytes, and necrotic keratinocytes in the epidermis layer [47]. This picture was not found in our study. However, apart from the findings of atypical keratinocytes, hyperkeratosis, and parakeratosis, in our study, an irregular dermoepidermal junction layer was found; this could be an early sign of further damage in the form of protruding shoot growth from the epidermis to the dermis, which is commonly found in precancerous lesions [48]. Of course, this still needs to be further proven by adding a longer treatment duration.

Furthermore, two issues should be acknowledged in regard to the treatment of subjects and the methodology used to collect histopathology data. This study falls short of addressing samples treated with only NB-UVB. The effect of tumor initiation remains unanswered as to whether it is caused by DMBA or NB-UVB. It is recommended for further study to include both separated treatments for each sample group. Second, a disadvantage of this framework was its inability to collect histopathology data after the DMBA application. Future study is suggested to provide different groups/samples for each DMBA time point.

## Conclusion

In conclusion, our study using a single dose of DMBA and 10 weeks of NB-UVB exposure in nongenetically modified Wistar rats demonstrates that this method can trigger changes indicative of the promotion phase of skin carcinogenesis. This approach revealed significant DNA damage, oxidative stress, and inflammation, accompanied by macroscopic and histological alterations consistent with early skin cancer development. This exposure increased CPD and MDA levels, indicating heightened DNA damage and oxidative stress, elevated pro-inflammatory cytokines (TNF-α, IL-6, and IL-11), and altered levels of regulatory cytokines (IL-10 and IL-12), highlighting the role of inflammation and immune modulation in tumor promotion. These molecular alterations were accompanied by macroscopic (erythema, skin thickening, desquamation, ulceration, and crusting) and histological changes (hyperkeratosis, epidermal thickening, and atypical keratinocytes), further indicating progression toward skin cancer promotion. However, this model mainly captures changes leading to the promotion phase and does not fully represent the entire carcinogenesis process. To thoroughly understand skin carcinogenesis, prolonged NB-UVB exposure is necessary. Future studies should extend the exposure period and include the additional control groups to gain clearer understanding of tumor progression and facilitate the development of effective preventive and therapeutic strategies against UVB-induced skin cancer.

## List of abbreviations

AK, actinic keratosis; BB-UVB, broadband ultraviolet B; CPD, cyclobutene pyrimidine dimer; cSCC, cutaneous squamous cell carcinoma; DMBA, dimethylbenzanthracene; HE, hematoxylin and eosin; IL, interleukin; MDA, malonedialdehyde; MED, minimal erythema dose; NFκB, nuclear factor kappa-B; NB-UVB, narrowband ultraviolet B; NMSC, nonmelanoma skin cancer; TBA, thiobarbituric acid; TBARS, thiobarbituric acid reactive substance; TNF-a, tumor necrosis factor a; TPA, 12-O-tetradecanoylphorbol-13-acetate.

## References

[1] Huang PY, Balmain A (2014). Modeling cutaneous squamous carcinoma development in the mouse. Cold Spring Harb Perspect Med.

[2] Di Nardo L, Pellegrini C, Di Stefani A, Del Regno L, Sollena P, Piccerillo A (2020). Molecular genetics of cutaneous squamous cell carcinoma: perspective for treatment strategies. J Eur Acad Dermatol Venereol.

[3] Kumah E, Bibee K (2023). Modelling cutaneous squamous cell carcinoma for laboratory research. Exp Dermatol.

[4] Howell JY, Ramsey ML (2023). Squamous cell skin cancer.

[5] Savoye I, Olsen CM, Whiteman DC, Bijon A, Wald L, Dartois L (2018). Patterns of ultraviolet radiation exposure and skin cancer risk: the E3N-SunExp study. J Epidemiol.

[6] Kennedy C, Willemze R, de Gruijl FR, Bouwes Bavinck JN, Bajdik CD (2003). The influence of painful sunburns and lifetime sun exposure on the risk of actinic keratoses, seborrheic warts, melanocytic nevi, atypical nevi, and skin cancer. J Invest Dermatol.

[7] Potenza C, Bernardini N, Balduzzi V, Losco L, Mambrin A, Marchesiello A (2018). A review of the literature of surgical and nonsurgical treatments of invasive squamous cells carcinoma. Biomed Res Int.

[8] Thomas G, Tuk B, Song JY, Truong H, Gerritsen HC, de Gruijl FR (2017). Studying skin tumourigenesis and progression in immunocompetent hairless SKH1-hr mice using chronic 7,12-dimethylbenz(a)anthracene topical applications to develop a useful experimental skin cancer model. Lab Anim.

[9] Quadri M, Marconi A, Sandhu SK, Kiss A, Efimova T, Palazzo E (2022). Investigating cutaneous squamous cell carcinoma *in vitro* and *in vivo*: novel 3d tools and animal models. Front Med.

[10] Kim I, He YY (2014). Ultraviolet radiation-induced non-melanoma skin cancer: regulation of DNA damage repair and inflammation. Genes Dis.

[11] Zhang X, Dwivedi C (2011). Skin cancer chemoprevention by a-santalol. Front Biosci.

[12] Ciążyńska M, Olejniczak-Staruch I, Sobolewska-Sztychny D, Narbutt J, Skibińska M, Lesiak A (2021). Ultraviolet radiation and chronic inflammation—molecules and mechanisms involved in skin carcinogenesis: a narrative review. Life.

[13] Bouceiro Mendes R, Alpalhão M, Filipe P (2022). UVB phototherapy in the treatment of vitiligo: state of the art and clinical perspectives. Photodermatol Photoimmunol Photomed.

[14] Mittal A, Kumar M, Gopishankar N, Kumar P, Verma AK (2021). Quantification of narrow band UVB radiation doses in phototherapy using diacetylene based film dosimeters. Sci Rep.

[15] Li Y, Cao Z, Guo J, Li Q, Zhu W, Kuang Y (2022). Assessment of efficacy and safety of uv-based therapy for psoriasis: a network meta-analysis of randomized controlled trials. Ann Med.

[16] Åkerla P, Pukkala E, Helminen M, Korhonen N, Karppinen T (2024). Skin cancer risk of narrow-band UV-B (TL-01) phototherapy: a multi-center registry study with 4,815 patients. Acta Derm Venereol.

[17] Kunisada M, Kumimoto H, Ishizaki K, Sakumi K, Nakabeppu Y, Nishigori C (2007). Narrow-band UVB induces more carcinogenic skin tumors than broad-band UVB through the formation of cyclobutane pyrimidine dimer. J Invest Dermatol.

[18] Yogianti F, Kunisada M, Ono R, Sakumi K, Nakabeppu Y, Nishigori C (2012). Skin tumours induced by narrowband UVB have higher frequency of p53 mutations than tumours induced by broadband UVB independent of Ogg1 genotype. Mutagenesis.

[19] Wan Mohammad WMZ (2017). Sample size calculation in animal studies using resource equation approach. Malays J Med Sci.

[20] Pakgohar A, Mehrannia H (2024). Sample size calculation in clinical trial and animal studies. Iranian J Diabetes Obes.

[21] Abel EL, Angel JM, Kiguchi K, DiGiovanni J (2009). Multi-stage chemical carcinogenesis in mouse skin: fundamentals and applications. Nat Protoc.

[22] Jeong WY, Kwon M, Choi HE, Kim KS (2021). Recent advances in transdermal drug delivery systems: a review. Biomater Res.

[23] Kapadia GJ, Azuine MA, Sridhar R, Okuda Y, Tsuruta A, Ichiishi E (2003). Chemoprevention of DMBA-induced UV-B promoted, NOR-1-induced TPA promoted skin carcinogenesis, and DEN-induced phenobarbital promoted liver tumors in mice by extract of beetroot. Pharmacol Res.

[24] de Jager TL, Cockrell AE, Du Plessis SS (2017). Ultraviolet light induced generation of reactive oxygen species. Adv Exp Med Biol.

[25] Premi S, Brash DE (2016). Chemical excitation of electrons: a dark path to melanoma. DNA Repair.

[26] Pfeifer GP (2020). Mechanisms of UV-induced mutations and skin cancer. Genome Instab Dis.

[27] Carpenter MA, Ginugu M, Khan S, Kemp MG (2022). DNA containing cyclobutane pyrimidine dimers is released from UVB-irradiated keratinocytes in a caspase-dependent manner. J Invest Dermatol.

[28] Toriyama E, Masuda H, Torii K, Ikumi K, Morita A (2021). Time kinetics of cyclobutane pyrimidine dimer formation by narrowband and broadband UVB irradiation. J Dermatol Sci.

[29] Gupta A, Avci P, Dai T, Huang YY, Hamblin MR (2013). Ultraviolet radiation in wound care: sterilization and stimulation. Adv Wound Care.

[30] Md Jaffri J (2023). Reactive oxygen species and antioxidant system in selected skin disorders. Malays J Med Sci.

[31] Terra VA, Souza-Neto FP, Pereira RC, Silva TNX, Costa ACC, Luiz RC (2012). Time-dependent reactive species formation and oxidative stress damage in the skin after UVB irradiation. J Photochem Photobiol B.

[32] Narendhirakannan RT, Hannah MAC (2013). Oxidative stress and skin cancer: an overview. Indian J Clin Biochem.

[33] Tran JT, Diaz MJ, Rodriguez D, Kleinberg G, Aflatooni S, Palreddy S (2023). Evidence-based utility of adjunct antioxidant supplementation for the prevention and treatment of dermatologic diseases: a comprehensive systematic review. Antioxidants.

[34] Divya MK, Salini S, Meera N, Lincy L, Seema M, Raghavamenon AC (2016). Attenuation of DMBA/croton oil induced mouse skin papilloma by *Apodytes dimidiata* mediated by its antioxidant and antimutagenic potential. Pharm Biol.

[35] Carrara IM, Melo GP, Bernardes SS, Neto FS, Ramalho LNZ, Marinello PC (2019). Looking beyond the skin: cutaneous and systemic oxidative stress in UVB-induced squamous cell carcinoma in hairless mice. J Photochem Photobiol B.

[36] Muqbil I, Azmi AS, Banu N (2006). Prior exposure to restraint stress enhances 7,12-dimethylbenz(*a*)anthracene (DMBA) induced DNA damage in rats. FEBS Lett.

[37] Hasan N, Nadaf A, Imran M, Jiba U, Sheikh A, Almalki WH (2023). Skin cancer: understanding the journey of transformation from conventional to advanced treatment approaches. Mol Cancer.

[38] Subhadarshani S, Athar M, Elmets CA (2020). Photocarcinogenesis. Curr Dermatol Rep.

[39] Zhang T, Ma C, Zhang Z, Zhang H, Hu H (2021). NF-κB signaling in inflammation and cancer. MedComm.

[40] Gunaseelan S, Balupillai A, Govindasamy K, Muthusamy G, Ramasamy K, Shanmugam M (2016). The preventive effect of linalool on acute and chronic UVB-mediated skin carcinogenesis in Swiss albino mice. Photochem Photobiol Sci.

[41] Sawicki K, Matysiak-Kucharek M, Kruszewski M, Wojtyła-Buciora P, Kapka-Skrzypczak L (2023). influence of chlorpyrifos exposure on UVB irradiation induced toxicity in human skin cells. J Occup Med Toxicol.

[42] Neagu M, Constantin C, Caruntu C, Dumitru C, Surcel M, Zurac S (2019). Inflammation: a key process in skin tumorigenesis (Review). Oncol Lett.

[43] Katiyar S (2007). Interleukin-12 and photocarcinogenesis. Toxicol Appl Pharmacol.

[44] Korinfsky JP, Plapler H, Moreno TR, Santos I, Maria C (2014). Induction of neoplastic cells in rat skin. Acta Cir Bras.

[45] Sharma SD, Katiyar SK (2010). Dietary grape seed proanthocyanidins inhibit UVB-induced cyclooxygenase-2 expression and other inflammatory mediators in UVB-exposed skin and skin tumors of SKH-1 hairless mice. Pharm Res.

[46] Rundhaug JE, Fischer SM (2010). Molecular mechanisms of mouse skin tumor promotion. Cancers.

[47] Goldenberg G, Golitz LE, Fitzpatrick J, Stockfleth E, Rosen T, Shumack S (2010). Histopathology of skin cancer. Managing skin cancer.

[48] Yanofsky VR, Mercer SE, Phelps RG (2011). Histopathological variants of cutaneous squamous cell carcinoma: a review. J Skin Cancer.

